# Reliability of Gemini 2.5 Pro, ChatGPT 4.1, DeepSeek V3, and Claude Opus 4 in generating standardized CMR protocols

**DOI:** 10.1186/s41747-025-00671-1

**Published:** 2026-01-26

**Authors:** Răzvan-Andrei Licu, Giuseppe Muscogiuri, Davide Casartelli, Anca Bacârea, Marian Pop, Andra-Maria Licu, Daniele Sferratore, Alessandro Caruso, Marianna Mirchuk, Piotr Tarkowski, Jakub Byczkowski, Sandro Sironi

**Affiliations:** 1 School of Medicine and Surgery, University of Milano-Bicocca, Milan, Italy; 2 Department of Radiology, ASST Papa Giovanni XXIII, Bergamo, Italy; 3 Doctoral School of Medicine and Pharmacy, George Emil Palade University of Medicine, Pharmacy, Science and Technology, Târgu Mureș, Romania; 4 Department of Radiology, County Emergency Clinical Hospital, Târgu Mureș, Romania; 5 Department of Pathophysiology, George Emil Palade University of Medicine, Pharmacy, Science and Technology, Târgu Mureș, Romania; 6 Faculty of Medicine in English, George Emil Palade University of Medicine, Pharmacy, Science and Technology, Târgu Mureș, Romania; 7 Department of Radiology, Dr. Fogolyán Kristóf County Emergency Hospital, Sfântu Gheorghe, Romania; 8 Department of Radiation Diagnostics, Danylo Halytsky Lviv National Medical University, Lviv, Ukraine; 9 Ukrainian-Polish Heart Center Lviv, Lviv, Ukraine; 10 Department of Radiology and Nuclear Medicine, University Hospital No 4 of Lublin, Lublin, Poland; 11 Department of Diagnostic Imaging, University Hospital No 1 of Lublin, Lublin, Poland; 12 Department of Radiology, Medical University of Gdańsk, Gdańsk, Poland

**Keywords:** Artificial intelligence, Cardiovascular disease, Large language models, Magnetic resonance imaging, Radiology

## Abstract

**Abstract:**

Artificial intelligence (AI) and large language models (LLMs) are increasingly integrated into radiology, offering new possibilities for advanced imaging techniques, including cardiovascular magnetic resonance (CMR). This proof-of-concept study assessed four high-performing LLMs (Gemini 2.5 Pro, ChatGPT 4.1, DeepSeek V3, and Claude Opus 4) on their ability to generate CMR protocols for 140 hypothetical cardiac cases. AI-generated protocols were compared against a reference standard established by a consensus between two experienced cardiovascular radiologists, following the Society for Cardiovascular Magnetic Resonance (SCMR) recommendations. Descriptive statistics were used to quantify the concordance of LLM-generated sequences with the SCMR guidelines. Statistical agreement was measured using Cohen and Fleiss κ statistics. Gemini 2.5 Pro achieved the highest concordance, aligning with the SCMR guidelines in 71.5% of all evaluated scenarios. Overall, LLMs showed moderate agreement with the SCMR protocols, with Gemini 2.5 Pro again performing best (Cohen κ = 0.55). Agreement was substantial for mandatory CMR sequences (Fleiss κ ∈ [0.69, 0.74]) and predominantly fair for optional sequences. The tested LLMs demonstrate a potential to generate efficient and pathology-adapted CMR protocols. Under expert supervision, this capability could streamline the imaging workflow and help extend CMR to primary healthcare centers through protocol automation.

**Relevance statement:**

The potential of Gemini 2.5 Pro, ChatGPT 4.1, DeepSeek V3, and Claude Opus 4 to suggest pathology-adapted CMR protocols could improve imaging throughput and help to expand access to advanced cardiac diagnostics in primary healthcare centers.

**Key Points:**

- The tested large language models show potential for generating CMR protocols.
- Substantial agreement on mandatory CMR sequences promises more efficient examinations.
- Automation of CMR protocols could help to improve access to this advanced technique outside major medical institutions.

**Graphical Abstract:**

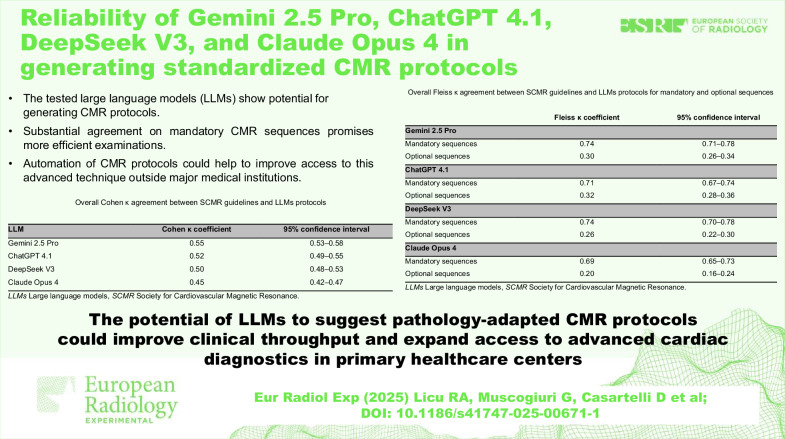

## Background

Artificial intelligence (AI) is represented by computing systems capable of performing tasks that traditionally require human intelligence, including learning, reasoning, pattern recognition, and decision-making [1, 2]. AI has undergone continuous and increasingly rapid improvement, especially through the evolution of deep learning algorithms and large-scale neural networks. These innovations are making their way into medical practice, in specialties such as radiology, offering potential benefits in optimizing image interpretation, workflow and providing diagnostic assistance [3, 4]. A breakthrough in AI is represented by large language models (LLMs) like Gemini, ChatGPT, DeepSeek, or Claude series [5–10].

Recently, cardiovascular magnetic resonance (CMR) has undergone significant advancements, with AI integration driving constant progress in diagnostic capabilities and workflow efficiency [11]. The role of CMR as a noninvasive technique that allows accurate analysis of anatomy, volumes and function in ischemic and non-ischemic cardiomyopathies has been well known in the literature [12]. In particular, late gadolinium enhancement, T1 and T2 mapping can be extremely helpful for the identification of underlying myocardial abnormalities and the potential role in terms of prognosis [13, 14]. Considering the pivotal role of CMR in clinical practice, scientific societies are standardizing the approach, adapting it to different scenarios [15–17].

AI applications are currently accelerating CMR workflow by shortening acquisition sequences and automating image post-processing. However, the potential for time reduction through pathology-specific protocol standardization and optimization using AI remains largely underexplored. To address this research gap, this proof-of-concept study assessed the possibility of building up CMR protocols using four high-performing LLMs (Gemini 2.5 Pro, ChatGPT 4.1, DeepSeek V3, and Claude Opus 4) across the spectrum of cardiac pathologies in comparison with protocols suggested by the Society for Cardiovascular Magnetic Resonance (SCMR).

## Materials and methods

**Fig. 1:**
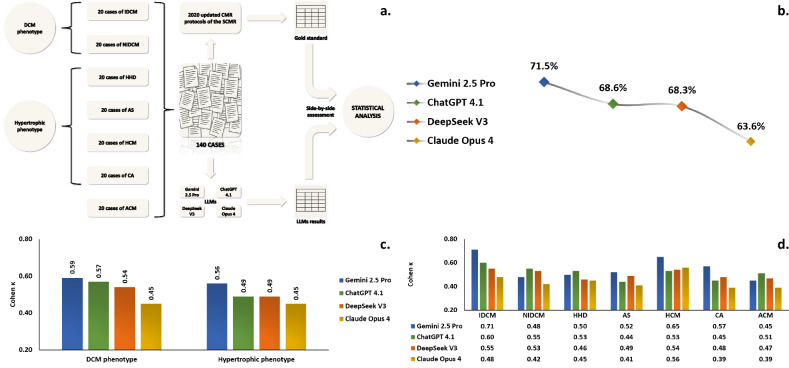
**a** Study design framework; **b** Overall concordance between SCMR guidelines and LLMs protocols; **c** Cohen κ agreement between SCMR guidelines and LLMs protocols by cardiac phenotype; **d** Cohen κ agreement between SCMR guidelines and LLMs protocols by cardiac pathology. ACM, Arrhythmogenic cardiomyopathy; AS, Aortic stenosis; CA, Cardiac amyloidosis; CMR, Cardiovascular magnetic resonance; DCM, Dilated cardiomyopathy; HCM, Hypertrophic cardiomyopathy; HHD, Hypertensive heart disease; IDCM, Ischemic dilated cardiomyopathy; LLMs, Large language models; NIDCM, Non-ischemic dilated cardiomyopathy; SCMR, Society for Cardiovascular Magnetic Resonance

Formal ethical approval was not required for this research, as the methodology was based on the analysis of simulated data and no human participants were involved. Figure 1a provides a detailed schematic representation, outlining the structure, components and progression of the research.

The study was conducted between June and August 2025 using a collection of comprehensive summaries for 140 hypothetical clinical scenarios, which were prospectively created by a radiologist with 5 years of expertise in cardiovascular imaging (see Supplementary Material). The dataset was evenly structured to represent a spectrum of cardiac diseases, including 20 patients with ischemic dilated cardiomyopathy (IDCM), 20 with non-ischemic dilated cardiomyopathy, 80 with hypertrophic phenotype, consisting of 20 cases each of hypertensive heart disease, aortic stenosis, hypertrophic cardiomyopathy (HCM) and cardiac amyloidosis, and an additional cohort of 20 patients with arrhythmogenic cardiomyopathy. The clinical scenario for each patient consisted of information on family history, personal medical background, relevant laboratory investigations, echocardiographic findings, and, when indicated, results from genetic testing. The rationale for creating our cases was to have a standardized and homogeneous cohort, which improves finding relevance and avoids misinterpretation. This approach also resolved data privacy concerns from using real-world patient data.

The personalized CMR scanning protocols for the created patients were designed by the cardiovascular radiologist with 5 years of experience in the field, then reviewed and approved by another radiologist with 12 years of expertise in cardiovascular imaging. The established CMR protocols comprised a set of mandatory and optional sequences and were fully compliant with the updated 2020 SCMR guidelines [16].

Hereafter, the radiologist with a 5-year background in cardiovascular imaging assessed each of the four LLMs tested by tasking them to generate patient-specific scanning protocols based on a single, direct zero-shot prompt. The question presented to LLMs was as follows: “According to the 2020 updated SCMR guidelines, which CMR imaging protocol do you recommend for this patient?”. The output was structured in tabular format, detailing both mandatory and optional imaging sequences to promote standardized formatting and improve the analytical utility of the results. Additionally, to prevent cross-case bias, a separate chat session was opened for each patient before querying AI models.

Statistical analysis was performed using JASP Version 0.19.3 (University of Amsterdam). The initial step in data analysis was to quantify how closely the sequences generated by LLMs aligned with those recommended in the SCMR guidelines. Afterward, Cohen κ coefficient was used to measure inter-rater reliability between our predefined gold standard, established in accordance with the 2020 SCMR criteria, and LLMs outputs. Data evaluation was performed first on the entire database, then on data stratified by patient phenotype. This stratified analysis focused on distinct subgroups, including patients with dilated cardiomyopathy (DCM) phenotype, classified as either IDCM or non-ischemic dilated cardiomyopathy, and those with hypertrophic phenotype (hypertensive heart disease, aortic stenosis, HCM, or cardiac amyloidosis). Subsequently, a systematic assessment was performed for each cardiac pathology. In the final phase of the analysis, Fleiss κ test was applied to highlight the level of agreement between guidelines and LLM-generated protocols when we strictly analyzed mandatory *versus* mandatory and optional *versus* optional sequences for overall cases. The resulting coefficients, both Cohen and Fleiss κ, were interpreted in line with the conventions summarized in Table 1, focusing on the magnitude of κ values and the precision of their 95% confidence intervals as measures of the agreement.

**Table 1:** Interpretation of Cohen and Fleiss κ coefficients

Value of κ coefficient	Strength of agreement
≤ 0	No agreement
0.01‒0.20	Slight
0.21‒0.40	Fair
0.41‒0.60	Moderate
0.61‒0.80	Substantial
0.81‒1.00	Almost perfect

See references [28], [29], and [30]

## Results

The primary outcome was the concordance between SCMR guidelines and LLM-suggested protocols over the 140 cases dataset. As presented in Fig. 1b, Gemini 2.5 Pro exhibited the highest concordance with 71.5%. ChatGPT 4.1, DeepSeek V3 and Claude Opus 4 followed with concordance values of 68.6%, 68.3% and 63.6%, respectively.

To formally quantify the level of agreement, inter-rater reliability between CMR protocols of the SCMR and AI-generated scanning protocols, treated as two raters, was assessed across all cases using Cohen κ coefficient. All four models showed moderate agreement with the guidelines, Gemini 2.5 Pro achieving the highest Cohen κ value of 0.55, though the performance of the other LLMs was comparable (Table 2).

**Table 2:** Overall Cohen κ agreement between SCMR guidelines and LLMs protocols

LLM	Cohen κ coefficient	95% confidence interval
Gemini 2.5 Pro	0.55	0.53‒0.58
ChatGPT 4.1	0.52	0.49‒0.55
DeepSeek V3	0.50	0.48‒0.53
Claude Opus 4	0.45	0.42‒0.47

*LLMs* Large language models, *SCMR* Society for Cardiovascular Magnetic Resonance

When patients were grouped by phenotype (Fig. 1c), a moderate level of agreement was again confirmed between SCMR guidelines and AI-generated protocols for both DCM and hypertrophic phenotypes. Cohen κ coefficient was slightly higher for Gemini 2.5 Pro (0.59 for DCM phenotype and 0.56 for hypertrophic phenotype), with comparable inter-rater agreement for ChatGPT 4.1 and DeepSeek V3 (0.57 and 0.54 for DCM phenotype, and both with 0.49 for hypertrophic phenotype). The lowest coefficients were recorded for Claude Opus 4, which scored 0.45 for both DCM and hypertrophic phenotypes.

Stratifying analysis by pathology allowed the assessment of agreement between standard SCMR protocols and LLM outcomes for each cardiac disease, the findings being detailed in Fig. 1d. Gemini 2.5 Pro demonstrated substantial agreement for IDCM and HCM, with Cohen κ values of 0.71 and 0.65, respectively. Shifting focus to the opposite end of the performance spectrum, Claude Opus 4 achieved fair agreement for cardiac amyloidosis and arrhythmogenic cardiomyopathy (Cohen κ = 0.39 for both pathologies). Regarding the remaining cases evaluated, a moderate level of agreement was observed, with Cohen κ values ranging from 0.41 for the aortic stenosis protocol generated by Claude Opus 4 to a borderline substantial agreement of 0.60 for the IDCM protocol suggested by ChatGPT 4.1.

The final analysis evaluated the agreement between guidelines and LLM-generated protocols by systematically assessing the mandatory and optional sequences defined by the raters for each CMR protocol (Table 3). Agreement was assessed using Fleiss κ test, which indicated substantial agreement with the SCMR guidelines among all four LLMs tested for mandatory sequences. Gemini 2.5 Pro and DeepSeek V3 achieved the highest Fleiss κ value of 0.74, followed by ChatGPT 4.1 (Fleiss κ = 0.71) and Claude Opus 4 (Fleiss κ = 0.69). Regarding optional sequences, Fleiss κ ranged from 0.20 for Claude Opus 4 to 0.32 for ChatGPT 4.1, suggesting a predominantly fair agreement between guidelines and LLMs protocols.

**Table 3:** Overall Fleiss κ agreement between SCMR guidelines and LLMs protocols for mandatory and optional sequences

	Fleiss κ coefficient	95% confidence interval
Gemini 2.5 Pro		
Mandatory sequences	0.74	0.71‒0.78
Optional sequences	0.30	0.26‒0.34
ChatGPT 4.1		
Mandatory sequences	0.71	0.67‒0.74
Optional sequences	0.32	0.28‒0.36
DeepSeek V3		
Mandatory sequences	0.74	0.70‒0.78
Optional sequences	0.26	0.22‒0.30
Claude Opus 4		
Mandatory sequences	0.69	0.65‒0.73
Optional sequences	0.20	0.16‒0.24

*LLMs* Large language models, *SCMR* Society for Cardiovascular Magnetic Resonance

## Discussion

Our study revealed the possibility of using LLMs for planning CMR protocols. Gemini 2.5 Pro performed best, matching SCMR guidelines in 71.5% of scenarios and achieving the highest overall moderate agreement (Cohen κ = 0.55). Gemini 2.5 Pro also reached substantial agreement for specific conditions, such as IDCM (Cohen κ = 0.71) and HCM (Cohen κ = 0.65), while other models showed moderate or fair agreement in some cases. Furthermore, all LLMs tested showed substantial agreement with the guidelines for mandatory CMR sequences (Fleiss κ ∈ [0.69, 0.74]) and only fair agreement for optional sequences. This suggests that LLMs can potentially generate efficient, pathology-targeted CMR protocols by prioritizing essential sequences. LLM-assisted CMR protocol aligns with recent studies supporting that LLMs can effectively identify appropriate cardiac imaging tests based on clinical presentation and societal guidelines [18]. Similarly, ChatGPT-4 demonstrated very high agreement with board-certified (neuro-) radiologists in selecting magnetic resonance imaging protocols, being able to suggest approved, time-saving options [19].

The findings of our study indicate a promising future for integrating LLMs into clinical applications of CMR. Under expert guidance, this could standardize and optimize protocols, decreasing workflow time, increasing daily scans and shortening waiting lists. By embedding a generative AI model into the routine workflow, another study found that radiologists can improve their documentation efficiency without compromising clinical quality [20]. The insights derived from our study also align with broader medical trends where LLMs, with oversight, enhance clinical practice. For instance, GPT-4 proved comparable to neuroradiologists in analyzing brain tumor magnetic resonance imaging reports [21]. Other LLMs analyzing BI-RADS showed moderate agreement with the human readers across multilingual reports [22]. In cardiology, ChatGPT-4.0 demonstrated high agreement with the physician-generated differential diagnoses, performing consistently regardless of case difficulty [23].

Another principal advantage of leveraging LLMs to generate CMR protocols is their potential to help extend this advanced imaging technique beyond major medical institutions, empowering primary healthcare centers that lack extensive expertise in this field. This concept is concurrently substantiated by existing AI applications proficient in sequence planning. Currently, one of the remaining prerequisites for achieving almost automated CMR acquisition is an AI technology capable of generating the optimal protocol for cardiac pathology, a capability that this study, through LLMs, demonstrates as feasible in the foreseeable future. Also, a study by Goh et al demonstrated that physicians utilizing an LLM achieved higher scores in managing complex clinical vignettes compared to those relying on conventional resources, although this advantage required more time per case. The work showed no significant difference between LLM-assisted physicians and LLM used alone [24]. Hence, through strategic implementation and clinical adoption of LLMs, medical practice and diagnostic capabilities could be improved, especially in settings with staffing shortages or less specialized medical professionals, such as rural areas.

While our study, together with other examples mentioned previously, indicates LLMs can assist clinicians, skepticism remains in the field of research. Critical studies cite inaccuracies compared to specialists in emergency medicine and oncology [25, 26], as well as risks of bias and misinformation [27]. Nevertheless, rapid AI progress suggests LLMs will become essential tools for physicians managing future healthcare demands, helping them optimize their daily workflow.

It is important to acknowledge that this study has some limitations. The tested LLMs lacked specific medical training, potentially impacting clinical reliability. Furthermore, our study used proprietary, “black box” models, which could hinder reproducibility and mean that our results may only reflect specific AI tools. Another limitation was the incomplete range of cardiac pathologies analyzed. For instance, the absence of a congenital heart disease subgroup may limit the generalizability of conclusions to all cardiac diseases. Moreover, in our attempt to simulate a real-time clinical query, our single-run design did not assess the LLM’s stochastic variability or output consistency, and this could be a key objective for future validation studies. Finally, performance was evaluated on synthetic cases due to data privacy concerns. The results obtained from these data may not translate directly to the complexities of daily medical practice, meaning clinical applicability therefore requires further validation with anonymized, real-world patient cases.

In conclusion, the tested LLMs consistently generated CMR protocols with moderate agreement to SCMR guidelines. Although agreement was substantial for mandatory sequences, suggesting potential to optimize efficiency, the overall performance highlights the need for a precautionary approach. With expert oversight, LLMs could help standardize workflow, reduce protocol time and expand CMR access through automation.

## Supplementary information

ELECTRONIC SUPPLEMENTARY MATERIAL

## Data Availability

The dataset analyzed during the current study is available in the Supplementary material.

## Abbreviations

AI: Artificial intelligence
CMR: Cardiovascular magnetic resonance
DCM: Dilated cardiomyopathy
HCM: Hypertrophic cardiomyopathy
IDCM: Ischemic dilated cardiomyopathy
LLMs: Large language models
SCMR: Society for Cardiovascular Magnetic Resonance
