# Supplementary material for: Reliability of Gemini 2.5 Pro, ChatGPT 4.1, DeepSeek V3, and Claude Opus 4 in generating standardized CMR protocols
Source: Eur Radiol Exp. 2026 Jan 26;10:7. doi: 10.1186/s41747-025-00671-1 (PMC12834875; doi:10.1186/s41747-025-00671-1)
Supplement: Supplementary file 1 — ELECTRONIC SUPPLEMENTARY MATERIAL [file 41747_2025_671_MOESM1_ESM.pdf]

Reliability of Gemini 2.5 Pro, ChatGPT 4.1, DeepSeek V3, and Claude Opus 4 in generating standardized CMR protocols

ELECTRONIC SUPPLEMENTARY MATERIAL

Clinical scenarios (*n* = 140) created by a radiologist with five years of expertise in cardiovascular imaging.

Abbreviations

AF Atrial fibrillation

AL Light-chain

ARVD Arrhythmogenic right ventricular dysplasia

AV Atrioventricular

AVA Aortic valve area

aVF Augmented unipolar limb lead in which the positive electrode is on the left leg

BAG3 Bcl-2-associated athanogene 3

BAV Bicuspid aortic valve

BNP B-type natriuretic peptide

BP Blood pressure

CABG Coronary artery bypass graft

CAD Coronary artery disease

CKD Chronic kidney disease

CK-MB Creatine kinase-myocardial band

COPD Chronic obstructive pulmonary disease

CRP C-reactive protein

DES Desmin

DSP Desmoplakin

ECG Electrocardiogram

EF Ejection fraction

eGFR Estimated glomerular filtration rate

FLNC Filamin-C

GLS Global longitudinal strain

HbA1c Glycated haemoglobin

HCM Hypertrophic cardiomyopathy

HFpEF Heart failure with preserved ejection fraction

HIV Human immunodeficiency virus

ICD Implantable cardioverter-defibrillator

IgA Immunoglobulin A

IgG Immunoglobulin G

JUP plakoglobin

LA Left atrium

LBBB Left bundle branch block

LDL Low-density lipoprotein

LMNA Lamin A/C

LV Left ventricle

LVEDD Left ventricular end-diastolic diameter

LVEF Left ventricular ejection fraction

LVH Left ventricular hypertrophy

LVOT Left ventricular outflow tract

M monoclonal

MI Myocardial infarction

MYBPC3 Cardiac myosin-binding protein C

MYH7 Myosin heavy chain 7

NT-proBPN N-terminal pro-B-type natriuretic peptide

NYHA New York Heart Association

PACs Premature atrial contractions

PKP2 Plakophilin-2

PLN Phospholamban

PVCs Premature ventricular contractions

QTc Corrected QT

RBBB Right bundle branch block

RV Right ventricle

SAM Systolic anterior motion

SCN5A Sodium channel, voltage gated, type V alpha subunit

Ser77Tyr Serine-to-tyrosine substitution at position 77

SPEP Serum protein electrophoresis

TAPSE Tricuspid annular plane systolic excursion

TAVI Transcatheter aortic valve implantation

TSH Thyroid-stimulating hormone

TTN Titin

TTR Transthyretin

UPEP Urine protein electrophoresis
Val122Ile Valine-to-isoleucine substitution
VT Ventricular tachycardia

1. A 65-year-old man with a long-standing history of hypertension and type 2 diabetes presents with progressive exertional dyspnea over the past year and occasional orthopnea. His father died of a myocardial infarction at age 58. Labs show stable, low-normal troponin levels, BNP elevated at 980 pg/mL, LDL cholesterol at 160 mg/dL, and HbA1c at 7.2%. The ECG reveals old Q waves in the inferior leads (II, III, aVF) and a left bundle branch block, suggesting prior infarction. Echocardiography shows a dilated left ventricle (LVEDD 65 mm), reduced ejection fraction at 30%, global hypokinesis, and mild secondary mitral regurgitation.

2. A 58-year-old woman with a 10-year history of stable angina now complains of gradually worsening fatigue and mild ankle edema over the past 6 months. She has no significant family history. Labs show normal troponin, BNP elevated at 450 pg/mL, and LDL at 130 mg/dL. Her ECG shows nonspecific ST depressions in lateral leads (V5-V6) and frequent premature ventricular contractions. Echocardiography reveals a dilated left ventricle (LVEDD 62 mm) with an ejection fraction of 35% and regional wall motion abnormality localized to the inferior wall, consistent with old ischemia.

3. A 72-year-old man with a 20-year history of coronary artery disease and prior myocardial infarction presents with worsening dyspnea on minimal exertion and episodes of palpitations. His brother also has ischemic heart disease. Labs show low-level troponin within chronic baseline, BNP elevated at 1,200 pg/mL, and LDL cholesterol at 180 mg/dL. ECG shows atrial fibrillation and deep Q waves in leads V1-V3, consistent with prior anterior infarction. Echocardiography reveals a reduced ejection fraction at 25%, a dilated LV (LVEDD 70 mm), apical akinesis, and moderate tricuspid regurgitation secondary to right heart strain.

4. A 60-year-old woman with a history of poorly controlled hypertension and stable coronary artery disease reports progressive breathlessness and orthopnea developing over several months. Her mother had heart failure. Lab results show negative troponin, BNP at 700 pg/mL, LDL at 140 mg/dL, and fasting glucose at 110 mg/dL. ECG reveals T wave inversions in the anterior leads and a prolonged QTc of 480 ms. Echocardiography shows a dilated left ventricle (LVEDD 60 mm) with an ejection fraction of 32% and anterior wall hypokinesis, suggestive of chronic ischemia.

5. A 67-year-old man with type 2 diabetes, hypertension, and known multi-vessel coronary artery disease presents with symptoms of chronic heart failure, including fatigue and paroxysmal nocturnal dyspnea. His son had early-onset coronary disease. Lab work shows stable low troponin, elevated BNP at 1,600 pg/mL, LDL cholesterol at 200 mg/dL, and poorly controlled diabetes with HbA1c at 8.1%. ECG reveals a left bundle branch block and signs of left atrial enlargement. Echocardiography demonstrates diffuse hypokinesis, a reduced ejection fraction of 28%, a dilated left ventricle (LVEDD 68 mm), and moderate mitral regurgitation.

6. A 55-year-old man with a history of early-onset coronary artery disease and smoking presents with worsening exertional dyspnea and reduced exercise tolerance over 8 months. His father had an MI at 50. Labs show elevated BNP at 1,100 pg/mL, LDL at 190 mg/dL, and normal troponin. ECG reveals deep Q waves in anterior leads and occasional ventricular ectopy. Echocardiography shows LV dilation (LVEDD 72 mm), ejection fraction at 22%, a large apical aneurysm, and an echogenic mural thrombus in the apex.

7. A 49-year-old woman with poorly controlled diabetes and prior silent myocardial infarction reports progressive shortness of breath and fatigue. No family history of CAD. Labs show BNP at 820 pg/mL, HbA1c 9.0%, and LDL at 170 mg/dL. ECG reveals poor R wave progression in precordial leads and left anterior fascicular block. Echocardiography demonstrates EF of 30%, moderate LV dilation, regional akinesis in the anterior and apical walls, and mild mitral regurgitation due to tethering.

8. A 63-year-old man with multivessel coronary artery disease and previous percutaneous interventions presents with worsening heart failure and orthopnea. Labs show BNP at 1,500 pg/mL, LDL at 160 mg/dL, and stable low troponin. His ECG shows atrial fibrillation and lateral T wave inversions. Echocardiography reveals EF of 28%, global hypokinesis, LVEDD 69 mm, moderate mitral regurgitation, and mild right ventricular dysfunction.

9. A 52-year-old man, an ex-smoker with a history of anterior myocardial infarction at age 45, complains of reduced exercise capacity and palpitations. Labs show BNP at 980 pg/mL, LDL at 180 mg/dL, and normal renal function. ECG shows frequent ventricular couplets and anterior Q waves. Echocardiography reveals an EF of 35%, dyskinetic anterior wall with aneurysmal bulging, and no thrombus. LV is dilated (LVEDD 65 mm).

10. A 70-year-old woman with long-standing hypertension and ischemic heart disease presents with worsening edema and fatigue. Labs show BNP at 2,000 pg/mL, LDL at 150 mg/dL. ECG reveals left bundle branch block and low voltage in limb leads. Echocardiography shows LV dilation (74 mm), EF of 20%, diffuse hypokinesis, severe functional mitral regurgitation, and mildly enlarged right atrium.

Eur Radiol (2025) Licu RA, Muscogiuri G, Casartelli D, et al.

11. A 48-year-old man with type 2 diabetes, hypertension, and a history of inferior myocardial infarction at 42 reports progressive exertional dyspnea and two episodes of near-syncope. BNP is 1,360 pg/mL, LDL 210 mg/dL, and HbA1c 8.7%. ECG shows old Q waves in II, III, aVF, with frequent non-sustained ventricular tachycardia runs. Echocardiography demonstrates EF 28%, a dilated LV (LVEDD 72 mm), inferior wall thinning with dyskinesis, and a small echogenic thrombus adherent to the inferoapical wall. There is moderate functional tricuspid regurgitation and mild pulmonary hypertension estimated at 45 mmHg.

12. A 54-year-old woman with stable angina in her 40s now has worsening NYHA class III heart failure symptoms and orthopnea. Her BNP is 980 pg/mL, LDL 170 mg/dL, and troponin is normal. ECG reveals left axis deviation and lateral T wave inversions. Echocardiography shows an ejection fraction of 33%, an LV dilation (LVEDD 62 mm), basal inferior wall hypokinesis, and moderate secondary mitral regurgitation with posterior leaflet tethering. The left atrium is dilated at 48 mm, consistent with longstanding pressure overload.

13. A 67-year-old man with multivessel coronary artery disease and prior stenting presents with worsening fatigue and recent onset of orthopnea. His labs show BNP 1,840 pg/mL, LDL 160 mg/dL. ECG shows atrial fibrillation with rapid ventricular response and prior anterior Q waves. Echocardiography demonstrates EF 25%, dilated LV (LVEDD 74 mm), akinesis of the entire anterior wall and apex, and moderate functional mitral regurgitation. There is also mild right ventricular dysfunction and early signs of hepatic congestion on abdominal ultrasound.

14. A 45-year-old man with known early-onset CAD and prior anterior MI presents with worsening exercise intolerance and occasional palpitations. Family history includes two brothers with premature CAD. Labs show BNP 1,020 pg/mL, LDL 200 mg/dL. ECG reveals Q waves in V1–V4 and frequent premature ventricular contractions. Echocardiography shows an ejection fraction of 30%, an aneurysmal dyskinetic anterior wall, LVEDD 68 mm, and no intracavitary thrombus. The mitral valve shows moderate regurgitation with posterior leaflet tethering. Strain imaging suggests severely reduced global longitudinal strain at −7%.

15. A 71-year-old woman with longstanding ischemic heart disease reports increasing orthopnea and leg swelling. BNP is markedly elevated at 2,200 pg/mL, LDL 150 mg/dL. ECG shows low voltage and left bundle branch block. On echocardiography, EF is 20%, with a dilated LV (LVEDD 76 mm), diffuse hypokinesis, severe functional mitral regurgitation due to leaflet tethering and annular dilation, and a small,

mobile apical thrombus. The right heart shows moderate enlargement with an estimated pulmonary artery systolic pressure of 50 mmHg, and there is mild pericardial effusion.

16. A 50-year-old man with prior silent myocardial infarction detected during a routine checkup now presents with NYHA class III symptoms and recent paroxysmal nocturnal dyspnea. His BNP is 1,400 pg/mL, LDL 185 mg/dL, and HbA1c 7.8%. ECG shows old Q waves in V2–V5 and frequent ventricular bigeminy. Echocardiography reveals an EF of 27%, LVEDD 70 mm, extensive anterior wall akinesis with a broad-based apical aneurysm. A large, laminated mural thrombus is seen at the apex, measuring approximately 15 mm in thickness. There is also moderate mitral regurgitation and left atrial enlargement (50 mm).

17. A 62-year-old woman with a history of stable angina now has progressive heart failure symptoms, including early satiety and abdominal bloating suggestive of right-sided involvement. BNP is 1,850 pg/mL, LDL 170 mg/dL. ECG shows atrial fibrillation with variable block and signs of prior inferior infarction. Echocardiography shows EF 24%, LVEDD 74 mm, severe global hypokinesis, moderate tricuspid regurgitation, and severely dilated right ventricle with reduced TAPSE (13 mm). Estimated pulmonary artery systolic pressure is 55 mmHg, and the inferior vena cava is dilated with reduced collapsibility.

18. A 46-year-old man with prior MI at 38 and multiple stents presents with worsening exercise intolerance and a recent near-syncope while walking. Family history is significant for premature CAD in both parents. BNP is 1,220 pg/mL, LDL 210 mg/dL. ECG shows Q waves in V1–V4, with frequent episodes of non-sustained ventricular tachycardia captured on Holter. Echocardiography demonstrates EF 30%, LVEDD 66 mm, large apical aneurysm, no thrombus, and severe reduction in longitudinal strain (−6%).

19. A 69-year-old man with chronic ischemic cardiomyopathy reports worsening dyspnea, significant lower limb edema, and episodes of lightheadedness. Labs reveal BNP 2,400 pg/mL and LDL 160 mg/dL. ECG shows left bundle branch block and left atrial enlargement. Echocardiography shows EF 22%, dilated LV (LVEDD 78 mm), diffuse hypokinesis, and large biventricular thrombi: one mural thrombus at the LV apex and another smaller thrombus in the right ventricle. Severe functional mitral and moderate tricuspid regurgitation are also present, along with early hepatic congestion seen on abdominal ultrasound.

20. A 73-year-old woman with ischemic cardiomyopathy and a remote history of anterior infarction presents after an episode of sustained ventricular tachycardia requiring emergency cardioversion. BNP is 2,100 pg/mL, LDL 150 mg/dL. Her ECG shows wide complex tachycardia with retrograde P waves, and baseline tracing shows old anterior Q waves and left bundle branch block. Echocardiography demonstrates an EF of 20%, LVEDD 76 mm, dyskinetic anterior and apical walls forming an aneurysmal

bulge, and moderate secondary mitral regurgitation. The right ventricle is borderline dilated but maintains preserved systolic function.

21. A 45-year-old male presented with exertional dyspnea and fatigue for six months. Family history was notable for sudden cardiac death in his father at age 50. NT-proBNP was 3,500 pg/mL. ECG showed sinus rhythm with LBBB (QRS 160 ms). Echocardiography revealed LVEF 25%, LVEDD 68 mm, and global hypokinesis. Holter monitoring showed non-sustained VT. Coronary angiography was normal. Genetic testing identified a truncating TTN mutation, consistent with familial non-ischemic dilated cardiomyopathy.

22. A 33-year-old female presented with palpitations and dizziness. Her brother had an ICD for ventricular arrhythmia. NT-proBNP was 1,800 pg/mL. ECG showed sinus rhythm with frequent premature ventricular contractions and lateral T-wave inversions. Echocardiography showed LVEF 30%, LVEDD 62 mm, and moderate mitral regurgitation. Holter confirmed paroxysmal VT. Coronary angiography was normal. Genetic testing revealed a likely pathogenic LMNA variant.

23. A 60-year-old male reported orthopnea, leg edema, and fatigue. His brother died of heart failure in his 50s. NT-proBNP was 5,600 pg/mL. ECG showed atrial fibrillation. Echocardiography revealed LVEF 20%, LVEDD 72 mm, and severe biatrial enlargement. A left ventricular thrombus was detected. Coronary angiography showed no disease. Genetic testing found a DSP gene mutation.

24. A 50-year-old female complained of reduced exercise tolerance. Family history included two maternal aunts with heart failure. NT-proBNP was 2,300 pg/mL. ECG showed low voltage and poor R-wave progression. Echocardiography revealed LVEF 35%, LVEDD 65 mm, and mild tricuspid regurgitation. Coronary angiography was normal. Genetic testing identified a BAG3 mutation.

25. A 28-year-old male collapsed during exercise. His father and uncle died young from sudden cardiac death. NT-proBNP was 950 pg/mL. ECG showed sinus rhythm with frequent premature ventricular contractions and non-sustained VT. Echocardiography revealed LVEF 40%, LVEDD 60 mm, and mild global hypokinesis. Coronary angiography was normal. Genetic testing showed a pathogenic SCN5A mutation.

26. A 52-year-old male presented with dyspnea on exertion and lower extremity edema. He reported heavy alcohol use for over 20 years, averaging more than 80 grams daily. NT-proBNP was 4,100 pg/mL. ECG showed sinus tachycardia with non-specific ST changes. Echocardiography revealed LVEF 28%, LVEDD 70 mm, and diffuse hypokinesis. No arrhythmia was seen on Holter. Coronary angiography was normal. Genetic testing was negative.

Eur Radiol (2025) Licu RA, Muscogiuri G, Casartelli D, et al.

27. A 39-year-old female presented with palpitations and near-syncope. Family history was unremarkable. NT-proBNP was 2,200 pg/mL. ECG showed sinus rhythm with first-degree AV block and frequent premature ventricular contractions. Echocardiography showed LVEF 32%, LVEDD 64 mm, and mild mitral regurgitation. Holter revealed runs of non-sustained VT. Coronary angiography was normal. Genetic testing found a likely pathogenic mutation in the FLNC gene.

28. A 48-year-old male with long-standing alcohol use reported worsening fatigue and early satiety. NT-proBNP was 3,300 pg/mL. ECG showed atrial fibrillation with a slow ventricular response. Echocardiography revealed LVEF 30%, LVEDD 68 mm, and moderate biatrial dilation. No thrombus was seen. Coronary angiography was normal. Genetic testing was not pursued.

29. A 55-year-old female presented with progressive dyspnea and orthopnea. She had no history of hypertension, diabetes, or CAD. Her mother was diagnosed with cardiomyopathy in her 60s. NT-proBNP was 2,800 pg/mL. ECG showed sinus rhythm with LBBB. Echocardiography showed LVEF 27%, LVEDD 66 mm, and moderate functional mitral regurgitation. Coronary angiography was normal. Genetic testing showed a truncating variant in the TTN gene.

30. A 42-year-old male experienced syncope while climbing stairs. His brother had a history of heart failure and ICD implantation. NT-proBNP was 2,900 pg/mL. ECG showed sinus rhythm with T-wave inversions in inferior leads. Echocardiography revealed LVEF 38%, LVEDD 61 mm, and mild RV dysfunction. Holter confirmed frequent premature ventricular contractions and non-sustained VT. Coronary angiography showed no disease. Genetic testing identified a pathogenic variant in the PLN gene.

31. A 47-year-old male presented with worsening dyspnea, abdominal distension, and peripheral edema. He had a history of chronic alcohol use but had stopped drinking six months earlier. NT-proBNP was 3,700 pg/mL. ECG showed atrial fibrillation with low voltage. Echocardiography revealed LVEF 30%, LVEDD 70 mm, and moderate RV dilation with reduced RV function. Mild tricuspid regurgitation and elevated pulmonary pressures were noted. Coronary angiography was normal. Genetic testing was not performed.

32. A 43-year-old female presented with fatigue and lightheadedness. There was no significant family history. NT-proBNP was 2,400 pg/mL. ECG showed sinus rhythm with frequent premature ventricular contractions. Echocardiography revealed LVEF 35%, LVEDD 63 mm, and mildly impaired RV function. Holter showed episodes of non-sustained VT. Coronary angiography showed normal coronary arteries. Genetic testing revealed a pathogenic variant in the DSP gene.

33. A 58-year-old male reported exertional dyspnea and leg swelling. Family history included a sister with heart failure. NT-proBNP was 4,900 pg/mL. ECG showed RBBB and atrial flutter. Echocardiography showed LVEF 28%, LVEDD 69 mm, and moderate RV systolic dysfunction. Severe tricuspid regurgitation and significant right atrial dilation were present. Coronary angiography was normal. Genetic testing revealed a truncating variant in the TTN gene.

34. A 41-year-old female with no comorbidities presented after an episode of syncope. NT-proBNP was 2,600 pg/mL. ECG showed sinus rhythm with epsilon wave-like notching in V1-V2. Echocardiography showed LVEF 36%, LVEDD 65 mm, and mild RV dysfunction. Holter revealed frequent premature ventricular contractions with a left bundle branch block morphology. Coronary angiography was normal. Genetic testing showed a pathogenic mutation in the PKP2 gene.

35. A 50-year-old male with dyspnea and early satiety was found to have ascites and peripheral edema. He had no alcohol use. NT-proBNP was 3,100 pg/mL. ECG showed sinus rhythm with low QRS voltage. Echocardiography demonstrated LVEF 34%, LVEDD 67 mm, moderate RV dysfunction, and moderate tricuspid regurgitation. No thrombus was noted. Coronary angiography showed no significant stenosis. Genetic testing revealed a likely pathogenic LMNA mutation.

36. A 46-year-old female presented with progressive dyspnea and palpitations. She had no cardiovascular risk factors, and her father was diagnosed with heart failure in his 50s. NT-proBNP was 2,800 pg/mL. ECG showed sinus rhythm with frequent premature ventricular contractions. Echocardiography revealed LVEF 33%, LVEDD 65 mm, and mild RV dysfunction. Holter confirmed non-sustained VT. Coronary angiography was normal. Genetic testing identified a pathogenic mutation in the BAG3 gene.

37. A 54-year-old male with a history of chronic alcohol use presented with worsening fatigue and abdominal bloating. NT-proBNP was 3,600 pg/mL. ECG showed atrial fibrillation and low voltage. Echocardiography showed LVEF 29%, LVEDD 68 mm, and moderate RV dysfunction with elevated right-sided pressures. Moderate tricuspid regurgitation was present. Coronary angiography was normal. Genetic testing was not pursued.

38. A 40-year-old female with no significant history presented after a syncopal episode. NT-proBNP was 1,900 pg/mL. ECG showed sinus rhythm with T-wave inversions in the lateral leads. Echocardiography revealed LVEF 38%, LVEDD 60 mm. Holter monitoring revealed frequent premature ventricular contractions and a short run of VT. Coronary angiography was normal. Genetic testing showed a pathogenic FLNC mutation.

39. A 49-year-old male reported worsening dyspnea and reduced exercise tolerance. Family history was positive for sudden cardiac death in two first-degree relatives. NT-proBNP was 4,200 pg/mL. ECG showed LBBB with QRS duration of 158 ms. Echocardiography revealed LVEF 26%, LVEDD 71 mm, and global hypokinesis. Mild RV dysfunction was noted. Coronary angiography was normal. Genetic testing revealed a truncating variant in the TTN gene.

40. A 44-year-old female presented with fatigue, early satiety, and lower limb swelling. NT-proBNP was 3,000 pg/mL. ECG showed sinus rhythm with low voltage QRS. Echocardiography showed LVEF 32%, LVEDD 66 mm, moderate RV dysfunction, and severe tricuspid regurgitation. A thrombus was seen in LV. Coronary angiography was normal. Genetic testing showed a likely pathogenic mutation in the DES gene.

41. A 58-year-old man with a 10-year history of poorly controlled hypertension presents with reduced exercise tolerance and mild dyspnea. His father had a stroke at 63 and his mother has long-standing hypertension. BP is 156/92 mmHg on lisinopril. ECG shows LVH with lateral ST-T changes. Laboratory results reveal normal renal function, normal glucose, and BNP 320 pg/mL. Echocardiography shows concentric LV hypertrophy (septum 14 mm), EF 60%, diastolic dysfunction, and mild left atrial enlargement.

42. A 60-year-old woman with well-controlled hypertension on amlodipine is seen for routine follow-up and is asymptomatic. Her sister has hypertensive heart disease, and her father had coronary artery disease. BP is 132/78 mmHg. ECG shows borderline LVH. Laboratory tests show NT-proBNP 160 pg/mL, normal renal function, and lipids at goal. Echocardiography reveals preserved EF (65%), diastolic dysfunction, and mild left atrial enlargement.

43. A 62-year-old man with long-standing hypertension and type 2 diabetes presents with fatigue and ankle edema. His brother has heart failure and CKD. BP is 148/90 mmHg; ECG reveals LVH with repolarization changes. Laboratory tests show creatinine 1.3 mg/dL, A1c 7.4%, and NT-proBNP 780 pg/mL. Echocardiography shows concentric hypertrophy (16 mm), EF 48%, left atrial enlargement, and diastolic dysfunction.

44. A 55-year-old man with long-standing hypertension complains of exertional dyspnea. His father had LV hypertrophy and stroke. BP is 162/95 mmHg on triple therapy. ECG shows marked LVH and left axis deviation. Laboratory tests reveal BNP 350 pg/mL and normal kidney function. Echocardiography shows severe concentric LVH (septum 18 mm), EF 65%, diastolic dysfunction, and significantly enlarged left atrium.

45. A 68-year-old woman with hypertension and obesity reports progressive dyspnea and nocturnal cough. Her mother had HFpEF and her sister has diabetes. BP is 150/88 mmHg. ECG shows atrial fibrillation with a controlled ventricular response. Laboratory tests show NT-proBNP 1,020 pg/mL, normal TSH, and slightly elevated CRP. Echocardiography reveals concentric LVH (14 mm), EF 60%, biatrial enlargement, and diastolic dysfunction. Chest x-ray shows pulmonary congestion.

46. A 72-year-old man with isolated systolic hypertension presents with palpitations. His mother had atrial fibrillation. BP is 160/74 mmHg. ECG shows sinus rhythm. Laboratory tests reveal NT-proBNP 270 pg/mL and normal kidney and thyroid function. Echocardiography shows preserved EF (66%), markedly enlarged left atrium, and impaired relaxation.

47. A 59-year-old woman with poorly controlled hypertension presents with palpitations and near-syncope. Her brother died suddenly at age 62. BP is 154/96 mmHg. ECG reveals frequent PVCs and nonspecific ST changes. Laboratory results show normal TSH, potassium, and magnesium. Holter shows > 2,000 PVCs/day. Echocardiography reveals concentric LVH (15 mm), EF 60%, and mild mitral regurgitation.

48. A 35-year-old man with newly diagnosed hypertension is referred after ECG showed LVH. His maternal grandfather had hypertrophic cardiomyopathy and both parents have hypertension. BP is 144/88 mmHg. ECG confirms LVH. Laboratory tests show NT-proBNP 110 pg/mL, normal creatinine, and a normal aldosterone/renin ratio. Echocardiography reveals septal wall thickness of 12 mm, EF 60%, with normal chamber size and no LVOT obstruction.

49. A 63-year-old woman with resistant hypertension reports mild exertional dyspnea. Her father had heart failure, and her mother had CKD. BP is 165/92 mmHg. ECG shows voltage criteria for LVH. Laboratory tests reveal eGFR 55 mL/min, glucose 102 mg/dL, troponin negative, and BNP 290 pg/mL. Echocardiography shows septal wall 14 mm, EF 62%, impaired relaxation, and reduced GLS (-14%).

50. A 66-year-old man with 20 years of hypertension presents with fatigue and ankle swelling. His brother has pulmonary hypertension, and his father had an MI at age 59. BP is 150/85 mmHg. ECG shows sinus rhythm with left atrial enlargement. Laboratory tests show BNP 240 pg/mL, HbA1c 6.1%, and normal renal profile. Echocardiography reveals concentric LVH (13 mm), EF 58%, mild tricuspid regurgitation, and an estimated pulmonary artery systolic pressure (PASP) of 38 mmHg.

51. A 48-year-old man with recently diagnosed hypertension presents with fatigue and mild chest tightness on exertion. His father had coronary artery disease and his sister was diagnosed with hypertension at 35. BP is 150/92 mmHg. ECG shows borderline LVH and early repolarization. Laboratory tests show normal

creatinine, glucose 98 mg/dL, and NT-proBNP 130 pg/mL. Echocardiography reveals mildly increased septal wall thickness (13 mm), normal chamber sizes including a non-dilated left atrium, and preserved EF (65%).

52. A 52-year-old woman with fluctuating blood pressure and anxiety-like symptoms presents with palpitations and episodic headaches. Her mother has hypothyroidism, and her father had hypertension. BP is 168/96 mmHg. ECG shows sinus tachycardia with a normal axis. Laboratory tests show normal renal panel, TSH 1.2, and plasma metanephrines pending. Echocardiography reveals normal LV wall thickness (10 mm), normal atrial size, and preserved EF. No significant valvular disease or diastolic dysfunction.

53. A 58-year-old male with a 15-year history of hypertension reports new fatigue and mild ankle swelling. His brother has hypertension, no family history of cardiac disease. BP is 145/88 mmHg. ECG shows LVH with secondary repolarization changes. Laboratory tests show NT-proBNP 210 pg/mL, creatinine 1.1 mg/dL, and sodium 137 mmol/L. Echocardiography reveals mildly increased LV mass index with relative wall thickness consistent with concentric remodeling, EF 58%, and a normal left atrial volume.

54. A 62-year-old woman with long-standing hypertension controlled on a thiazide presents with episodes of dizziness. Her father had atrial fibrillation but no structural heart disease. BP is 140/85 mmHg. ECG shows sinus bradycardia and prolonged PR interval. Laboratory tests show potassium 3.4 mmol/L, magnesium 1.8 mg/dL, and BNP 105 pg/mL. Echocardiography shows a small LV cavity with concentric wall thickening (septum 13 mm), normal atrial size, and EF 66%. No significant valvular abnormalities or diastolic dysfunction.

55. A 64-year-old male with intermittent BP spikes is referred for cardiac evaluation due to exertional fatigue. His mother had diabetes, father unknown. BP is 158/94 mmHg. ECG shows normal sinus rhythm with slight left axis deviation. Laboratory tests show glucose 112 mg/dL, creatinine 1.2 mg/dL, aldosterone/renin ratio elevated. Echocardiography reveals LV wall thickness at upper normal limits (11 mm), normal LV volume and LA size, EF 62%.

56. A 45-year-old woman with newly diagnosed hypertension presents with sleep disturbances and a dry cough. Her mother has asthma, no cardiac family history. BP is 152/86 mmHg. ECG is normal. Laboratory tests show mildly elevated CRP, NT-proBNP 180 pg/mL, and normal creatinine. Echocardiography shows a non-dilated LV with borderline increased wall thickness, preserved systolic function, and normal left atrial volume index. No valvular disease or filling abnormalities.

57. A 59-year-old male with poorly documented hypertension presents with chest heaviness and lightheadedness. His father had an MI at 62. BP is 142/90 mmHg. ECG reveals PVCs and a borderline QTc interval. Laboratory tests show normal renal panel and electrolytes, BNP 160 pg/mL. Echocardiography shows LV septal wall at 13 mm, EF 55%, no left atrial enlargement, and mild mitral annular calcification. Global longitudinal strain is at the lower limit of normal (-16%).

58. A 51-year-old woman with borderline hypertension and a history of migraines presents with occasional palpitations. Her brother has high blood pressure, otherwise unremarkable family history. BP is 146/88 mmHg. ECG shows frequent PACs with normal intervals. Laboratory tests show NT-proBNP 90 pg/mL, normal thyroid and kidney function. Echocardiography reveals a normal LV size and function, trace concentric thickening (septum 13 mm), and normal left atrial volume. Doppler parameters suggest early-stage diastolic impairment without elevation in filling pressures.

59. A 67-year-old man with 20 years of mild hypertension reports reduced exercise stamina but no dyspnea. His brother had stroke at 70. BP is 148/82 mmHg. ECG shows old LBBB. Laboratory tests reveal BNP 200 pg/mL and eGFR 60. Echocardiography shows preserved EF (57%), borderline concentric LVH (12 mm), and normal atrial size. RV function is normal. No regional wall motion abnormalities or pulmonary hypertension.

60. A 55-year-old woman with newly diagnosed hypertension and no prior cardiac history presents for baseline evaluation. Her father had hypertension and CKD. BP is 150/90 mmHg. ECG shows borderline LVH. Laboratory tests include normal creatinine, NT-proBNP 140 pg/mL, and aldosterone slightly elevated. Echocardiography demonstrates normal cavity size, preserved EF, concentric remodeling with increased relative wall thickness, and a normal LA volume index.

61. A 68-year-old male with a history of hypertension and hyperlipidemia presents with progressive exertional dyspnea and occasional dizziness over the past 6 months. His father underwent valve replacement at age 70. On examination, he has a late-peaking systolic ejection murmur best heard at the right upper sternal border, radiating to the carotids. BP is 138/74 mmHg. ECG reveals LVH with repolarization abnormalities. Echocardiography is inconclusive for the assessment of the aortic valve. The LV is hypertrophied with preserved EF (60%) and no regional wall motion abnormalities. BNP is elevated at 520 pg/mL.

62. A 73-year-old woman with type 2 diabetes and moderate CKD presents with fatigue and reduced functional capacity (NYHA class III). Her mother had aortic stenosis requiring surgery at age 75. On auscultation, a crescendo-decrescendo murmur is heard in the second intercostal space with diminished

second heart sound. BP is 132/80 mmHg. ECG shows sinus rhythm with LVH. Transthoracic echocardiography is inconclusive for the assessment of the aortic valve, showing an EF 52%. There is mild concentric hypertrophy and mild diastolic dysfunction. NT-proBNP is 860 pg/mL, and creatinine is 1.4 mg/dL.

63. A 60-year-old man presents with progression of symptoms of chest heaviness and presyncope. His brother had BAV and underwent valve replacement at 58. On exam, a harsh systolic murmur radiates to the neck, and a soft diastolic murmur is audible as well. BP is 142/78 mmHg. ECG shows LVH with repolarization changes. Echocardiography is inconclusive for the assessment of the aortic valve due to difficult window. EF is 58%, with asymmetric septal hypertrophy. BNP is 410 pg/mL.

64. A 75-year-old female with a background of hypertension and COPD presents with worsening dyspnea and fatigue and has a longstanding systolic murmur. No family history of valvular disease. BP is 140/76 mmHg. ECG shows normal sinus rhythm with LVH voltage. Echocardiography reveals moderate aortic stenosis (peak velocity 3.1 m/s, mean gradient 28 mmHg) with preserved EF (65%) and mild LV concentric remodeling. The AVA is measured at 1.2 cm^2, but image quality is limited by body habitus and COPD-related hyperinflation. NT-proBNP is 690 pg/mL.

65. A 66-year-old man with rheumatoid arthritis and long-standing hypertension presents with exertional dyspnea and lightheadedness. There is no family history of cardiac disease, but he has a long-standing ejection systolic murmur. BP is 145/85 mmHg. ECG shows left axis deviation and anterior T wave inversions. Echocardiography is inconclusive for the assessment of the aortic valve but reveals a concentric hypertrophy and preserved EF. Laboratory tests show NT-proBNP 740 pg/mL and troponin elevated at 0.03 ng/mL. There is suspicion of infiltrative pathology.

66. A 71-year-old man with a history of transcatheter aortic valve implantation 18 months ago for severe calcific aortic stenosis presents with increasing dyspnea and atypical chest pressure. There is no family history of valve disease. On exam, a soft systolic murmur is present. BP is 132/80 mmHg. ECG shows sinus rhythm with left anterior fascicular block. Echocardiography reveals a functional bioprosthesis but with rising gradients and trace paravalvular leak. EF is 52%.

67. A 28-year-old male with known congenital unicuspid aortic valve presents with exertional chest discomfort and dizziness. His father had congenital heart disease but no valve history. A harsh systolic murmur is noted along the left sternal border. BP is 124/76 mmHg. ECG reveals LVH with ST depressions in V5-V6. Echocardiography shows severe aortic stenosis (peak velocity 4.5 m/s, mean gradient 42 mmHg, AVA 0.7 cm^2), EF 64%, and mild ascending aorta dilation. BNP is 280 pg/mL.

68. A 69-year-old woman with paradoxical low-flow, low-gradient aortic stenosis and preserved EF presents for second opinion after inconclusive echocardiography. No family history of cardiac disease. BP is 140/72 mmHg. ECG shows LVH. Echocardiography shows AVA 0.75 cm², mean gradient 26 mmHg, and LVEF 53% with small LV cavity and concentric remodeling. NT-proBNP is 670 pg/mL.

69. A 75-year-old man with COPD and prior CABG presents with worsening dyspnea and borderline hypotension. Family history is irrelevant. The exam reveals diminished heart sounds and crackles breath sounds. ECG shows Q waves in the anterior leads. Echocardiography rises suspicion of severe aortic stenosis (AVA 0.6 cm²), mean gradient 45 mmHg, and EF 38%. There's uncertainty whether symptoms are valve-related or due to ischemic dysfunction. BNP is 890 pg/mL, troponin mildly elevated.

70. A 35-year-old woman with Turner syndrome and prior bicuspid aortic valve repair presents with fatigue and occasional palpitations. Her mother has hypertension but no structural disease. BP is 118/70 mmHg. ECG shows incomplete RBBB and sinus rhythm. Echocardiography reveals moderate aortic stenosis (peak velocity 3.3 m/s) and ascending aorta dilation (43 mm). EF is normal. BNP is 180 pg/mL.

71. A 76-year-old male with severe aortic stenosis and EF 35% presents for TAVI candidacy evaluation. He reports fatigue, orthopnea, and unintentional weight loss. No family history of cardiomyopathy. BP is 135/78 mmHg. ECG shows LBBB. Echocardiography demonstrates heavily calcified tricuspid aortic valve, AVA 0.6 cm², peak velocity 4.7 m/s, and concentric LV hypertrophy. NT-proBNP is 2,100 pg/mL.

72. A 42-year-old male athlete presents with effort intolerance and vague chest tightness. He has no history of cardiac diseases. BP is 128/74 mmHg. ECG shows early repolarization. Echocardiography reveals a possible bicuspid aortic valve, with mild aortic stenosis (peak 2.8 m/s), borderline septal thickening (12 mm), and mildly dilated aortic root. BNP is 110 pg/mL.

73. A 68-year-old woman with rheumatoid arthritis presents with progressive shortness of breath and lightheadedness. No known cardiac family history. BP is 140/80 mmHg. ECG shows low voltages and first-degree AV block. Echocardiography demonstrates moderate aortic stenosis (AVA 1.1 cm²), EF 54%, and mild concentric hypertrophy. There is suspicion of infiltrative pathology. NT-proBNP is 920 pg/mL.

74. A 63-year-old male with hypertension and CKD presents with fatigue and mild cognitive slowing. His father died of sudden cardiac death at 60. BP is 142/86 mmHg. ECG reveals LVH with lateral repolarization abnormalities. Echocardiography shows AVA 1.0 cm², mean gradient 40 mmHg, and asymmetric septal hypertrophy. EF is preserved. BNP is 410 pg/mL.

75. A 70-year-old man with a bioprosthetic surgical aortic valve implanted 10 years ago presents with fatigue and new-onset atrial fibrillation. No family history of valve disease. BP is 135/78 mmHg. ECG confirms AF with controlled rate. Echocardiography shows increased gradients across the prosthesis (mean 34 mmHg), normal EF, and mild RV enlargement. BNP is 530 pg/mL.

76. A 61-year-old woman presents with dyspnea, edema, and recent weight gain. She has no known valve history. Her sister has heart failure with preserved EF. BP is 140/84 mmHg. ECG shows normal sinus rhythm with low voltage QRS. Echocardiography suggests moderate aortic stenosis (peak 3.2 m/s, AVA 1.1 cm^2) and concentric hypertrophy. EF is preserved, LA size is borderline. BNP is 760 pg/mL.

77. A 30-year-old male with coarctation repair in childhood presents with fatigue and upper limb hypertension. His mother had bicuspid aortic valve. BP is 150/80 mmHg in right arm. ECG shows LVH with T wave inversion. Echocardiography suspects bicuspid valve with moderate aortic stenosis and ascending aorta dilation (45 mm). BNP is 260 pg/mL.

78. A 69-year-old woman with mild cognitive impairment and multiple falls is found to have severe aortic stenosis during geriatric assessment. Family history is unavailable. BP is 130/70 mmHg. ECG shows sinus bradycardia and low voltages. Echocardiography shows heavily calcified aortic valve, AVA 0.65 cm^2, mean gradient 39 mmHg, EF 50%, and small LV cavity. NT-proBNP is 850 pg/mL.

79. A 62-year-old male with HIV presents with fatigue and chest discomfort. His father had heart failure of unknown cause. BP is 138/76 mmHg. ECG shows normal sinus rhythm with premature ventricular beats. Echocardiography shows moderate aortic stenosis with valve thickening and mild aortic regurgitation. EF is 55%, with mild septal thickening. BNP is 400 pg/mL. There is suspicion of HIV-associated cardiomyopathy.

80. A 74-year-old male with treated Hodgkin lymphoma (chest radiation 25 years ago) presents with increasing fatigue and dyspnea. No cardiac family history. BP is 132/78 mmHg. ECG reveals low voltages and left axis deviation. Echocardiography shows moderate-to-severe aortic stenosis with heavily calcified valve, AVA 0.9 cm^2, preserved EF, and evidence of mild pericardial thickening. BNP is 640 pg/mL. There is suspicion of radiation-induced heart disease.

81. A 28-year-old man presents with exertional dyspnea and near-syncope during a soccer match. His father died suddenly at age 42 with no known cardiac history. On the physical exam, there is a systolic ejection murmur at the left sternal border that increases with Valsalva. BP is 124/76 mmHg. ECG shows

left ventricular hypertrophy with deep T wave inversions in the lateral leads. Laboratory tests reveal NT-proBNP 360 pg/mL and high-sensitivity troponin within normal limits. Transthoracic echocardiography shows asymmetric septal hypertrophy (septum 18 mm, posterior wall 11 mm), preserved EF (70%), systolic anterior motion (SAM) of the mitral valve, and a resting LVOT gradient of 35 mmHg.

82. A 42-year-old woman with a history of mild hypertension reports occasional palpitations and one episode of presyncope while climbing stairs. Her brother has an ICD for HCM. BP is 130/78 mmHg. ECG shows sinus rhythm with deep T wave inversions in V4-V6 and borderline QTc prolongation. NT-proBNP is 280 pg/mL, potassium 4.1 mmol/L. Echocardiography reveals preserved EF (65%), moderate asymmetric septal thickening (17 mm), and no resting gradient. LV cavity is mildly reduced, with preserved diastolic function.

83. A 50-year-old male with type 2 diabetes and no prior cardiac history presents with exertional chest tightness and shortness of breath. His uncle had unexplained heart failure in his 50s. BP is 140/84 mmHg. ECG shows LVH and left axis deviation. Troponin is negative, NT-proBNP 420 pg/mL, HbA1c 7.1%. Echocardiography reveals concentric LV thickening (septum 15 mm, posterior wall 14 mm), small LV cavity, and no significant outflow gradient. EF is 68%, and no valvular abnormalities are noted.

84. A 33-year-old asymptomatic woman is referred after her genetic testing revealed a pathogenic MYBPC3 mutation during a workup for her father’s sudden cardiac death. Her clinical exam is normal, and BP is 120/76 mmHg. ECG shows sinus rhythm with mild Q waves in inferior leads. Laboratory tests are normal, including NT-proBNP 95 pg/mL. Transthoracic echocardiography shows normal LV wall thickness (10 mm), normal diastolic function, and no LVOT gradient or valvular abnormalities.

85. A 38-year-old male with a history of frequent premature ventricular contractions and occasional palpitations presents for evaluation. His mother has HCM and an ICD. BP is 128/80 mmHg. ECG shows frequent PVCs and T wave inversion in the anterolateral leads. Holter reveals 5,200 PVCs/day and short runs of non-sustained VT. NT-proBNP is 380 pg/mL. Transthoracic echocardiography shows mild asymmetric septal thickening (14 mm), preserved EF, and no obstruction at rest or with provocation.

86. A 40-year-old man with hypertension and increasing dyspnea is referred after an abnormal ECG. His father had HCM and underwent ICD implantation. BP is 134/82 mmHg. ECG shows deep T wave inversions in leads V4-V6 and LVH. NT-proBNP is 470 pg/mL. Echocardiography reveals asymmetric septal hypertrophy (17 mm), preserved EF, and dynamic LVOT gradient of 42 mmHg at rest. Mild mitral regurgitation is present due to SAM.

87. A 52-year-old woman with known hypertension and palpitations is evaluated after an episode of near-syncope. Her maternal uncle had sudden cardiac death. BP is 138/84 mmHg. ECG shows normal sinus rhythm with Q waves in lateral leads. Holter monitoring reveals non-sustained VT. NT-proBNP is 560 pg/mL. Echocardiography shows asymmetric septal hypertrophy (16 mm), EF 62%, and no outflow obstruction. Genetic tests are positive for pathogenic mutation for HCM.

88. A 36-year-old asymptomatic woman with a MYH7 mutation identified during family screening presents for baseline imaging. Her father had HCM and died suddenly at age 49. BP is 118/72 mmHg. ECG shows sinus rhythm with increased QRS voltage and no repolarization abnormalities. NT-proBNP is 120 pg/mL. Transthoracic echocardiography shows borderline septal thickness (12 mm), no gradient, and normal EF.

89. A 29-year-old male recreational runner reports palpitations and vague chest discomfort. No family history of cardiomyopathy or sudden death. BP is 122/74 mmHg. ECG shows high-voltage QRS and T wave inversions in V5-V6. NT-proBNP is 240 pg/mL. Echocardiography shows asymmetric hypertrophy (14 mm) with preserved EF and no obstruction.

90. A 62-year-old woman with fatigue and exertional dyspnea is referred for evaluation of suspected apical HCM. Family history is negative. BP is 140/86 mmHg. ECG shows giant negative T waves in precordial leads. NT-proBNP is 610 pg/mL. Echocardiography reveals increased apical wall thickness (15 mm), normal EF, and no obstruction.

91. A 47-year-old man with paroxysmal atrial fibrillation and HCM family history presents with reduced exercise tolerance. BP is 130/80 mmHg. ECG shows AF with controlled ventricular response. Echocardiography reveals septal wall thickness of 17 mm, normal EF, no obstruction, and mild LA enlargement. NT-proBNP is 680 pg/mL.

92. A 33-year-old woman experiences exertional syncope during hiking. Her brother had HCM and underwent septal myectomy. BP is 126/78 mmHg. ECG shows LVH with lateral ST depression. Troponin is negative. NT-proBNP 430 pg/mL. Echocardiography shows dynamic LVOT obstruction (resting gradient 36 mmHg, increasing to 78 mmHg with Valsalva), EF 65%, and SAM.

93. A 45-year-old male with type 2 diabetes and well-controlled hypertension presents with palpitations and borderline LV wall thickening on echocardiography. His father had diastolic heart failure. BP is 138/84 mmHg. ECG shows sinus rhythm and left axis deviation. NT-proBNP is 360 pg/mL. Echocardiography shows symmetric thickening (13 mm septum/posterior), small LV cavity, and diastolic dysfunction.

94. A 31-year-old asymptomatic male with borderline ECG changes is referred after routine screening due to family history of HCM in a cousin. NT-proBNP is 145 pg/mL. Echocardiography shows wall thickness at 13 mm, no gradient, and preserved systolic and diastolic function.

95. A 54-year-old woman with HCM diagnosed 5 years ago presents with new chest pain and worsening fatigue. No family history of coronary disease. BP is 128/80 mmHg. ECG shows normal sinus rhythm with lateral ST-T changes. Troponin is normal. BNP 720 pg/mL. Echocardiography shows stable septal hypertrophy (16 mm), mild mitral regurgitation, and EF 58%.

96. A 38-year-old man presents with palpitations and is found to have frequent PVCs and short non-sustained ventricular tachycardia episodes on Holter. His father has an ICD for HCM. BP is 130/82 mmHg. ECG shows sinus rhythm with anterior T wave inversion. Echocardiography reveals moderate septal hypertrophy (16 mm), no gradient, and preserved EF. NT-proBNP is 410 pg/mL.

97. A 41-year-old woman with mild hypertension and a murmur noted in pregnancy presents for cardiac workup. Her uncle died suddenly at 45. BP is 138/88 mmHg. ECG shows mild LVH. NT-proBNP is 190 pg/mL. Echocardiography shows mild asymmetric hypertrophy (13 mm septum), preserved EF, and no obstruction.

98. A 61-year-old man with known HCM and progressive dyspnea reports new exertional chest pain. His brother died suddenly in his 40s. BP is 134/80 mmHg. ECG shows LVH with inferolateral T wave inversion. NT-proBNP is 840 pg/mL. Echocardiography shows EF 60%, septal thickness 19 mm, and no significant LVOT obstruction.

99. A 26-year-old female athlete is referred for abnormal ECG during pre-participation screening. Her mother is healthy, father unknown. BP is 114/70 mmHg. ECG shows giant negative T waves in anterolateral leads and LVH. NT-proBNP is 180 pg/mL. Echocardiography reveals preserved EF, apical wall thickening (13 mm), and small LV cavity.

100. A 44-year-old man with moderate exertional dyspnea, palpitations, and elevated BP presents for evaluation. His mother had unexplained cardiac arrest. BP is 142/88 mmHg. ECG reveals normal sinus rhythm with signs of LVH and ST depressions. NT-proBNP is 640 pg/mL. Echocardiography shows asymmetric septal hypertrophy (16 mm), normal EF, and no resting obstruction.

101. A 74-year-old man with a history of carpal tunnel syndrome and spinal stenosis presents with fatigue and exertional dyspnea. His father had late-onset heart failure and died in his 80s. On exam, he has mild

bilateral ankle edema and a low-volume carotid pulse. BP is 128/72 mmHg. ECG shows low-voltage QRS in limb leads and pseudo-infarct pattern in anterior precordial leads. Echocardiography reveals concentric LV thickening (septum 16 mm), sparkling myocardial texture, EF 50%, and diastolic dysfunction. NT-proBNP is 2,100 pg/mL. Troponin T is 0.04 ng/mL. Serum free light chain assay is normal, and serum protein electrophoresis (SPEP)/urine protein electrophoresis (UPEP) are negative. Hematology consultation found no evidence of plasma cell dyscrasia.

102. A 63-year-old female with no prior cardiac history presents with progressive fatigue and orthostatic hypotension. Her brother has multiple myeloma. On the physical exam, she has macroglossia, periorbital purpura, and mild peripheral neuropathy. BP is 108/70 mmHg supine, dropping to 86/58 mmHg on standing. ECG shows low voltage and first-degree AV block. Echocardiography reveals thickened LV walls (septum 15 mm), small LV cavity, preserved EF, and severe diastolic dysfunction. NT-proBNP is 2,950 pg/mL. Troponin I is 0.07 ng/mL. Serum free light chain ratio is abnormal (kappa/lambda = 0.12), and SPEP shows a faint monoclonal spike. Hematology confirms IgG lambda monoclonal gammopathy, bone marrow biopsy shows 12% plasma cells.

103. A 69-year-old man with chronic kidney disease and bilateral shoulder pain presents with worsening dyspnea and leg swelling. No family history of cardiomyopathy. Exam reveals jugular venous distension, hepatomegaly, and bilateral crackles. BP is 134/82 mmHg. ECG shows normal sinus rhythm with low voltages and diffuse T wave flattening. Echocardiography demonstrates biventricular wall thickening, EF 45%, restrictive filling pattern, and biatrial enlargement. NT-proBNP is 3,200 pg/mL. Troponin T is 0.05 ng/mL. Serum and urine electrophoresis are negative, and free light chains are within normal range. Hematology suggests no evidence of clonal disorder.

104. A 55-year-old man with a family history of “nerve disease” presents with palpitations, weight loss, and orthostatic symptoms. His father died in his 60s after years of neuropathy and heart failure. BP is 116/76 mmHg, falling to 90/60 mmHg on standing. ECG shows low-voltage QRS and right bundle branch block. Echocardiography reveals concentric LV hypertrophy (septum 14 mm), normal EF, and restrictive filling. NT-proBNP is 1,750 pg/mL. Genetic testing is pending for transthyretin mutations. Serum free light chain assay is normal. Hematology excluded plasma cell dyscrasia.

105. A 66-year-old woman with atrial fibrillation and unexplained heart failure with preserved EF is referred for evaluation of possible infiltrative disease. Her brother has polyneuropathy and a pacemaker. On physical exam, she has mild periorbital bruising, irregular rhythm, and trace edema. BP is 130/78 mmHg. ECG shows AF with low voltages. Echocardiography shows moderate LV wall thickening (septum 15 mm), EF 55%, mild mitral regurgitation, and biatrial enlargement. NT-proBNP is 2,500 pg/mL. Troponin I is 0.06

ng/mL. Free light chain assay is borderline elevated (lambda 35 mg/L, kappa 15 mg/L), with equivocal SPEP. Hematology is performing bone marrow biopsy and fat pad aspirate.

106. A 76-year-old male with long-standing carpal tunnel syndrome and lumbar spinal stenosis presents with increasing fatigue and mild leg swelling. His brother was diagnosed with heart failure at age 80. BP is 134/76 mmHg. ECG shows low-voltage QRS and pseudoinfarct pattern in V1-V3. Echocardiography reveals concentric LV thickening (septum 17 mm), EF 52%, and restrictive diastolic filling. NT-proBNP is 2,300 pg/mL. Troponin T is 0.05 ng/mL. Serum free light chains and SPEP/UPEP are negative.

107. A 64-year-old female with type 2 diabetes and chronic diarrhea presents with worsening fatigue and hypotension. She reports numbness in her feet and unintentional weight loss. BP is 100/64 mmHg. ECG shows low-voltage QRS and first-degree AV block. Echocardiography shows thickened LV walls (15 mm), EF 50%, biatrial enlargement, and mitral annular calcification. NT-proBNP is 2,900 pg/mL. Troponin I is 0.08 ng/mL. Serum free light chains reveal elevated lambda 42 mg/L, kappa 14 mg/L, with abnormal ratio. SPEP shows a monoclonal spike. Hematology confirms AL amyloidosis.

108. A 69-year-old man with a recent diagnosis of bilateral carpal tunnel syndrome and sensorimotor neuropathy presents with fatigue and dyspnea on exertion. His father had late-onset heart failure. BP is 126/74 mmHg. ECG shows sinus rhythm with low voltages. Echocardiography reveals concentric LVH (septum 16 mm), EF 56%, and diastolic dysfunction. NT-proBNP is 1,880 pg/mL. Free light chains are normal, and SPEP/UPEP are unremarkable. Genetic testing reveals a pathogenic TTR Val122Ile mutation.

109. A 55-year-old man with chronic inflammatory arthritis presents with exertional dyspnea and peripheral edema. No family history of amyloidosis. BP is 122/70 mmHg. ECG shows low voltages and a prolonged PR interval. Echocardiography shows symmetric biventricular thickening, EF 48%, and mild pericardial effusion. NT-proBNP is 2,400 pg/mL. Troponin T is 0.06 ng/mL. Serum free light chains are normal, SPEP negative.

110. A 72-year-old woman with recurrent atrial fibrillation and unexplained LV hypertrophy is referred for advanced imaging. Her brother had cardiac amyloidosis. BP is 138/80 mmHg. ECG shows AF with low voltages and poor R wave progression. Echocardiography reveals concentric thickening (septum 14 mm), EF 60%, and biatrial enlargement. NT-proBNP is 2,150 pg/mL. Free light chains are mildly elevated, but ratio is within normal limits. SPEP shows faint M-spike. Hematology work-up is inconclusive.

111. A 68-year-old male with CKD and prior stroke presents with fatigue and reduced exercise tolerance. His family history includes a brother with renal failure. BP is 136/82 mmHg. ECG shows sinus rhythm with

low-voltage limb leads. Echocardiography demonstrates thickened septum (16 mm), reduced LV compliance, and EF 58%. NT-proBNP is 1,750 pg/mL, creatinine 2.1 mg/dL. Free light chain ratio is abnormal (kappa 10, lambda 55). Hematology suspects AL amyloidosis with renal and cardiac involvement.

112. A 63-year-old man presents with worsening dyspnea and orthostatic hypotension. He reports a 10-kg weight loss, chronic paresthesia, and erectile dysfunction. No cardiac family history. BP is 110/70 mmHg. ECG shows low voltages and frequent PVCs. Echocardiography shows LV wall thickness of 15 mm, preserved EF, and mild RV involvement. NT-proBNP is 2,600 pg/mL. Free light chain assay shows lambda 60 mg/L, kappa 18 mg/L, ratio 0.3. SPEP positive for IgG-lambda. Bone marrow shows 15% plasma cells.

113. A 74-year-old woman with a history of heart failure with preserved EF is referred for evaluation after a recent diagnosis of spinal stenosis and bilateral carpal tunnel syndrome. Her father had heart disease in late life. BP is 128/76 mmHg. ECG shows normal sinus rhythm with low voltages. Echocardiography reveals septal thickness of 16 mm, EF 55%, and moderate diastolic dysfunction. NT-proBNP is 1,980 pg/mL. Free light chain profile and SPEP are normal. Bone scintigraphy is pending.

114. A 59-year-old male with newly diagnosed nephrotic-range proteinuria and peripheral edema presents with progressive fatigue and palpitations. His uncle died of kidney failure in his 60s. BP is 124/76 mmHg. ECG reveals sinus rhythm with low voltages and left atrial enlargement. Echocardiography shows thickened LV walls (septum 15 mm), EF 62%, and mild pericardial effusion. NT-proBNP is 2,750 pg/mL, creatinine 1.6 mg/dL. SPEP shows monoclonal protein, free light chain ratio is abnormal. Hematology confirms systemic AL amyloidosis.

115. A 60-year-old man with history of atrial flutter ablation and HFpEF is referred due to progressive fatigue and wall thickening on echocardiography. His father had a pacemaker and unexplained bradycardia. BP is 130/78 mmHg. ECG shows sinus rhythm with low voltages and prolonged PR interval. Echocardiography demonstrates septal thickness 15 mm, normal EF, restrictive filling, and biatrial dilation. NT-proBNP is 2,300 pg/mL. Free light chains are normal, SPEP negative. Genetic testing reveals TTR Ser77Tyr variant.

116. A 43-year-old man with progressive neuropathy, erectile dysfunction, and weight loss presents with new-onset exertional dyspnea. His father was diagnosed with "nerve disease" in his 50s. BP is 118/74 mmHg with an orthostatic drop to 92/62 mmHg. ECG shows sinus rhythm with low-voltage limb leads. Echocardiography reveals septal thickening (14 mm), EF 58%, and a mildly reduced global longitudinal

strain with apical sparing. NT-proBNP is 1,850 pg/mL. SPEP and free light chains are normal. Genetic test is inconclusive.

117. A 38-year-old woman with systemic lupus erythematosus presents with unexplained dyspnea and syncope. Family history is negative. BP is 122/70 mmHg. ECG shows sinus rhythm with borderline low voltages and PR prolongation. Echocardiography reveals concentric thickening (septum 13 mm), EF 60%, and moderate biatrial enlargement. NT-proBNP is 1,400 pg/mL. Troponin I is mildly elevated. Free light chain ratio is mildly abnormal (kappa 12, lambda 34). Hematology considers AL amyloidosis vs inflammatory myocardial disease.

118. A 45-year-old male presents with new-onset atrial fibrillation and unexplained concentric LV hypertrophy. His mother had heart failure of unknown cause in her 60s. BP is 128/78 mmHg. ECG confirms AF with low-voltage QRS complexes. Echocardiography reveals septal wall thickness of 15 mm, EF 57%, and trace pericardial effusion. NT-proBNP is 2,300 pg/mL. Free light chains and SPEP are unremarkable.

119. A 50-year-old man with IgA nephropathy and recent development of peripheral neuropathy presents with fatigue, dizziness, and reduced effort tolerance. Family history is irrelevant. BP is 118/76 mmHg. ECG shows normal sinus rhythm with low-voltage limb leads. Echocardiography shows symmetric LV thickening (septum 15 mm), EF 60%, and mild mitral regurgitation. NT-proBNP is 2,600 pg/mL. Free light chain ratio is borderline abnormal, and SPEP shows a faint monoclonal spike. Hematology suspects systemic AL amyloidosis with renal and cardiac involvement.

120. A 46-year-old woman with idiopathic polyneuropathy, postural dizziness, and unintentional weight loss is referred for evaluation of possible infiltrative cardiomyopathy. Her father had cardiac symptoms in his 50s. BP is 116/72 mmHg with orthostatic hypotension. ECG shows sinus rhythm with low voltage. Echocardiography shows borderline concentric thickening (septum 12 mm), EF 59%, and impaired longitudinal strain with apical sparing. NT-proBNP is 1,670 pg/mL. Genetic testing is pending. Light chains are normal.

121. A 22-year-old male athlete was admitted after a syncopal episode during training, with a three-month history of palpitations and lightheadedness. Family history included sudden cardiac death in a paternal uncle at age 35 and frequent ventricular ectopy in his father. Troponin and CK-MB were normal, BNP was mildly elevated at 180 pg/mL, and genetic testing revealed a PKP2 mutation. ECG showed T-wave inversions in V1-V3, an epsilon wave in V1, incomplete RBBB, and frequent premature ventricular contractions with LBBB morphology and inferior axis. Echocardiography demonstrated mild RV dilation

and regional hypokinesia. Holter monitoring recorded over 5,000 premature ventricular contractions in 24 hours and several episodes of nonsustained VT.

122. A 36-year-old woman reported palpitations and one presyncopal episode. Her sister had cardiomyopathy, and her father received an ICD at 48. Troponin and electrolytes were normal. BNP 135 pg/mL. DSP gene variant identified. ECG showed T-wave inversions in V1-V4 and frequent premature ventricular contractions with LBBB morphology. Echocardiography revealed mild RV enlargement and subtle wall motion abnormalities. Holter recorded over 3,500 premature ventricular contractions and short runs of nonsustained VT.

123. A 17-year-old male collapsed during a football game. He had a prior history of exercise-related skipped beats. A cousin died suddenly at 20. Troponin was borderline elevated. ECG showed epsilon wave in V2, T-wave inversions in V1-V3, and VT with LBBB morphology. Echocardiography revealed moderate RV dilation with reduced function. Holter showed frequent premature ventricular contractions and nonsustained VT.

124. A 40-year-old woman was admitted with recurrent wide-complex tachycardia requiring cardioversion. She had months of fatigue and palpitations. Her mother died suddenly at 45. Troponin was mildly elevated, magnesium low-normal. The genetic test was negative. ECG showed T-wave inversions V1-V5 and frequent premature ventricular contractions. Echocardiography showed RV systolic dysfunction. Holter captured both sustained and nonsustained VT.

125. A 29-year-old asymptomatic male was referred after premature ventricular contractions were found on routine screening. His brother has an ICD for ARVD. Troponin normal, BNP 105 pg/mL. JUP mutation was detected. ECG showed isolated premature ventricular contractions with LBBB pattern and T-wave inversions in V1-V2. Echocardiography showed mild RV dilation and outflow tract hypokinesia. Holter recorded over 2,500 premature ventricular contractions.

126. A 21-year-old male presented after a brief loss of consciousness during a basketball game. No prior cardiac history. His maternal uncle died suddenly in his early 30s. Routine labs were unremarkable. ECG showed frequent premature ventricular contractions with left bundle branch block morphology and T-wave inversions in V1-V3. Echocardiography revealed right ventricular enlargement with mildly reduced contractility. A 48-hour Holter confirmed multiple runs of nonsustained ventricular tachycardia and more than 6,000 premature ventricular contractions.

127. A 34-year-old woman was referred for evaluation of palpitations and exertional dizziness. Her brother has an ICD and was previously diagnosed with a genetic cardiomyopathy. Troponin and BNP were within normal limits. ECG showed inverted T waves in V1-V4 and intermittent premature ventricular contractions. Genetic testing revealed a pathogenic PKP2 variant. Echocardiography showed borderline right ventricular size with focal hypokinesia. Holter monitoring documented over 4,000 premature ventricular contractions and several brief episodes of nonsustained VT.

128. A 25-year-old man, recreational runner, was evaluated for persistent palpitations and occasional chest discomfort. He had no known family history of heart disease. Cardiac enzymes were normal. ECG revealed frequent premature ventricular contractions with left bundle branch block configuration, and T-wave inversions limited to V1-V2. Echocardiography showed subtle hypokinesia of the RV free wall. Holter showed over 3,000 premature ventricular contractions without sustained arrhythmia. Genetic testing was inconclusive.

129. A 31-year-old female experienced nocturnal palpitations and one episode of near-syncope. Her mother died suddenly at age 50. Troponin normal, with BNP slightly elevated. ECG demonstrated frequent premature ventricular contractions with left bundle branch block morphology and an epsilon wave in V1. Echocardiography revealed regional wall motion abnormality of the right ventricle with preserved global function. A 24-hour Holter recorded over 5,000 premature ventricular contractions and one 12-beat run of nonsustained VT.

130. A 19-year-old male collapsed during track practice and recovered quickly. His father has a history of ventricular arrhythmias. ECG showed T-wave inversions in right precordial leads and frequent premature ventricular contractions with inferior axis. BNP and troponin were normal. Echocardiography showed mildly dilated right ventricle with hypokinetic areas along the outflow tract. Holter monitoring identified over 7,000 premature ventricular contractions and two episodes of nonsustained VT.

131. A 32-year-old woman presented with exertional palpitations and one episode of syncope while climbing stairs. Her younger brother has a known diagnosis of arrhythmogenic cardiomyopathy. Troponin was normal. BNP was 165 pg/mL. ECG showed frequent premature ventricular contractions with right bundle branch block morphology and T-wave inversions in the lateral leads. Echocardiography demonstrated mild dilation of the left ventricle with regional hypokinesia in the inferolateral wall and preserved right ventricular function. Holter monitoring detected over 6,000 premature ventricular contractions, several with polymorphic features, and two brief runs of nonsustained VT. Genetic testing revealed a desmoplakin (DSP) mutation.

132. A 27-year-old male reported progressive fatigue and palpitations over six months. There was no family history of heart disease. Lab tests were unremarkable. ECG showed low QRS voltage in limb leads and frequent premature ventricular contractions with alternating bundle branch morphologies. Echocardiography revealed moderate right ventricular dilation and mild systolic dysfunction, as well as patchy hypokinesia in the lateral wall of the left ventricle. Holter confirmed over 8,000 premature ventricular contractions and frequent ventricular couplets. Genetic testing was negative.

133. A 24-year-old man presented after a collapse during recreational cycling. He had no previous symptoms. His father died suddenly at age 40. Troponin was normal, BNP was elevated at 200 pg/mL. ECG revealed T-wave inversions in V1-V3, an epsilon wave in V2, and frequent premature ventricular contractions with left bundle branch block morphology. Echocardiography showed moderate right ventricular dysfunction and borderline enlargement of the left ventricle with preserved ejection fraction. Holter monitoring documented over 9,000 premature ventricular contractions and several nonsustained VT episodes.

134. A 30-year-old woman complained of irregular heartbeat and brief dizziness during rest. Family history was unremarkable. Troponin and BNP were within normal limits. ECG showed frequent premature ventricular contractions with right bundle branch block morphology and T-wave inversions in the inferolateral leads. Echocardiography showed normal right ventricular size but akinesia in the mid-lateral segment of the left ventricle. Holter recorded over 4,000 premature ventricular contractions. A plakophilin-2 gene mutation was detected.

135. A 20-year-old male was referred after being found to have frequent ventricular ectopy during military enlistment screening. He was asymptomatic. His mother has a defibrillator for an unspecified cardiomyopathy. Lab tests were normal. ECG showed frequent premature ventricular contractions with left bundle branch block morphology and an epsilon wave in lead V1. Echocardiography demonstrated mild right ventricular dilation and reduced right ventricle EF, with normal left ventricular parameters. Holter monitoring showed over 5,000 premature ventricular contractions in 24 hours, some occurring in bigeminy.

136. A 26-year-old man presented with sudden onset of chest fluttering during rest. He had no prior cardiac complaints. His father had an ICD placed for ventricular arrhythmias. Troponin and BNP were normal. ECG showed frequent premature ventricular contractions with left bundle branch block morphology and T-wave inversions in V1-V2. Echocardiography revealed mildly enlarged right ventricle with localized hypokinesia. Left ventricular function was preserved. Holter monitoring recorded over 7,000 premature ventricular contractions, mostly monomorphic, and short runs of nonsustained VT.

137. A 33-year-old woman was evaluated for intermittent palpitations and reduced exercise tolerance. She denied syncope. Family history included a sister with a cardiomyopathy of unknown type. Troponin normal, BNP mildly elevated. ECG showed T-wave inversions in V1-V3 and frequent premature ventricular contractions with right bundle branch block morphology. Echocardiography revealed left ventricular dilation with reduced ejection fraction (45%) and regional akinesia in the basal inferolateral segment. The right ventricle appeared structurally normal. Holter recorded over 4,000 premature ventricular contractions with occasional ventricular couplets.

138. A 30-year-old male truck driver reported occasional palpitations and near-fainting episodes while seated. No cardiac history. A cousin died suddenly at 27. ECG showed low voltage QRS complexes in limb leads and frequent premature ventricular contractions with left bundle branch block morphology and superior axis. Echocardiography revealed right ventricular dilation with focal aneurysm formation, and normal left ventricular size. Holter documented over 6,000 premature ventricular contractions and multiple short runs of nonsustained VT. Genetic testing identified a variant in the desmoglein-2 (DSG2) gene.

139. A 22-year-old competitive swimmer collapsed after a race and recovered spontaneously. Family history notable for a paternal aunt with a defibrillator. ECG showed epsilon wave in V1, T-wave inversions in V1-V4, and frequent premature ventricular contractions with left bundle branch block morphology. BNP was 190 pg/mL. Echocardiography demonstrated moderate right ventricular dysfunction and regional akinesia in the anterior wall of the left ventricle. Holter monitoring confirmed over 8,000 premature ventricular contractions and episodes of nonsustained VT.

140. A 31-year-old female complained of skipped beats and fatigue, more noticeable in the evenings. No family history of heart disease. Troponin was normal, BNP was 150 pg/mL. ECG revealed frequent premature ventricular contractions with right bundle branch block morphology and negative T waves in leads V5-V6. Echocardiogram showed normal right ventricle but patchy left ventricular hypokinesia, especially in the lateral segments. Holter detected over 3,000 premature ventricular contractions and one run of nonsustained VT lasting 11 beats.
